# Study of Optical Configurations for Multiple Enhancement of Microalgal Biomass Production

**DOI:** 10.1038/s41598-018-38118-w

**Published:** 2019-02-11

**Authors:** Changsoon Cho, Kibok Nam, Yeong Hwan Seo, Kyoohyun Kim, YongKeun Park, Jong-In Han, Jung-Yong Lee

**Affiliations:** 1 Graduate School of Energy, Environment, Water, and Sustainability (EEWS), Korea Advanced Institute of Science and Technology (KAIST), Daejeon, 34141 Republic of Korea; 2 Department of Chemical and Biomolecular Engineering, Korea Advanced Institute of Science and Technology (KAIST), Daejeon, 34141 Republic of Korea; 3 Department of Civil and Environmental Engineering, Korea Advanced Institute of Science and Technology (KAIST), Daejeon, 34141 Republic of Korea; 4 Present Address: Agency for Defense Development, Daejeon, 34188 Republic of Korea; 5 Department of Physics, Korea Advanced Institute of Science and Technology (KAIST), Daejeon, 34141 Republic of Korea; 6 Present Address: Biotechnology Center, Technische Universität Dresden, 01307 Dresden, Germany; 7 Tomocube Inc., Daejeon, 34051 Republic of Korea

## Abstract

Microalga is a promising biomass feedstock to restore the global carbon balance and produce sustainable bioenergy. However, the present biomass productivity of microalgae is not high enough to be marketable mainly because of the inefficient utilization of solar energy. Here, we study optical engineering strategies to lead to a breakthrough in the biomass productivity and photosynthesis efficiency of a microalgae cultivation system. Our innovative optical system modelling reveals the theoretical potential (>100 g m^−2^ day^−1^) of the biomass productivity and it is used to compare the optical aspects of various photobioreactor designs previously proposed. Based on the optical analysis, the optimized V-shaped configuration experimentally demonstrates an enhancement of biomass productivity from 20.7 m^−2^ day^−1^ to 52.0 g m^−2^ day^−1^, under the solar-simulating illumination of 7.2 kWh m^−2^ day^−1^, through the dilution and trapping of incident energy. The importance of quantitative optical study for microalgal photosynthesis is clearly exhibited with practical demonstration of the doubled light utilization efficiencies.

## Introduction

Photosynthesis is the principle process by which life converts solar energy and CO_2_ into reduced and functional carbon forms and is the product of billions of years of evolution. The global photosynthetic rate of ~130 TW^1,2^ secures environmental homeostasis by maintaining the carbon balance between land and atmosphere. However, since the industrial revolution, humans have increasingly rapidly burned carbon chemicals (~16 TW)^3^ accumulated over the last 100 million years, causing carbon imbalance and, consequently, increasingly daunting global climate change on Earth. Therefore, renewable energy alternatives must be developed and implemented in a multilateral and unceasing manner^4–7^. The depletion of the finite chemical energy resources is another reason to pursue such alternatives, especially because of the necessity of carbon-based liquid fuels for transportation (~4 TW)^8^ at least for the near future. Biofuels are generally viewed as a solution: they are produced in a continuous manner and reduce CO_2_ in the process. However, this potentially green solution, particularly to become effectively commercialized, has many issues that must be overcome. The most important issue is the requirement for large amounts of arable land: at least 6 more Amazon rainforests^9–11^ are required to meet the 4 TW demand through the cultivation of terrestrial plants such as grains and trees.

One promising alternative to terrestrial biomass feedstock is microalgal biomass. These phototrophic microorganisms achieve a 10- to 50-fold higher photosynthesis rate (PR) than terrestrial plants^12–14^; therefore, they need a far smaller land area for biomass production than their terrestrial counterparts. Nevertheless, there exist plenty of challenges for production of microalgal biomass with monoculture, such as avoiding contamination, enhancing lipid contents, and reducing production cost. In particular, we focus on the fact that the present biomass productivity of 10–20 g m^−2^ day^−1^ in open-pond cultivation systems, which are advantageous for scaling up and for mass production, is far from profitable, especially in the form of fuels^12–15^. Given that such a low productivity has much do with the exceedingly limited utilization of the incoming light, ingenious optical engineering can offer a breakthrough in tackling this otherwise almost insurmountable challenge. Previous attempts can broadly be classified into two: (i) quantity control for diluting strong incident light energy with light guides^16–22^, vertically or obliquely installed reactors^23–26^, tubular or spiral design^27–33^, or increased surface areas^34–37^; and (ii) quality control for effectively utilizing the solar spectrum with luminescent materials^38–46^. Although these efforts are useful in various ways, the comprehension of optical behavior of microalgae in particular is greatly lacking, which fundamentally hampers design innovation able to overcome such limited performance.

The present study aims to make the best of the optical engineering for the purpose of maximizing the biological counterpart, namely, microalgal photosynthesis; and in so doing, establishing general and specific design rules encompassing economically viable optical strategies in a way that extracts the full potential of microalgal biomass productivity. Based on a 3D profile analysis for refractive indices of algal cells, a realistic model for photosynthetic systems is proposed to better understand the macroscopic behaviour of the photosynthetic microbes. The theoretical analysis predicts that biomass productivity can reach ~140 g m^−2^ day^−1^ by way of light energy redistribution under high illumination. To realize this theoretical potential in a practical sense, which is directly applicable to an open pond, a V-shaped cultivator is chosen. When the light energy is efficiently diluted and trapped by adopting the V-shaped bioreactor under an illumination of 7.2 kWh m^−2^ day^−1^, the biomass productivity is experimentally shown to be improved more than 2.5-fold, from 20.7 g m^−2^ day^−1^ to 52.0 g m^−2^ day^−1^.

## Results and Discussion

### Experimental design

For the microalgal research, the large-scale outdoor cultivation and lab-scale indoor evaluation have strong pros and cons of each. The outdoor cultivation provides the same environmental condition as the real world application and it is considered to be more trustworthy in the industry; however, its environmental dependence, uncontrollability, and high installation cost have been a high barrier to entry for scientific investigation and innovative challenges. On the other hand, despite the convenience and controllability, lab-scale indoor experiments have been considered to be rarely reproducible in the real-world. Therefore, this research has focused on the establishment of a rigorous outdoor-simulating cultivation system, which would bring the system design work to the field of academic research. A metal halide lamp with proper spectral filters (K3700, McScience, Korea) was adopted to simulate outdoor conditions. Within the wavelength range of 400–800 nm, the relative portions of the photons in 400–500 nm, 500–600 nm, 600–700 nm and 700–800 nm were 28%, 27%, 29%, and 16%, on average, while those are 26%, 28%, 26%, and 21% in the real sunlight with the spectrum of AM 1.5 G, exhibiting only a reasonable deviation of a few percent in the visible range. The illumination intensities were maintained within 0.55–0.60 *sun* by using a photodiode detecting 300–800 nm, where 1 *sun* intensity corresponds to 1000 W m^−2^ for AM 1.5 G and visible photons of 2000 μmol m^−2^ s^−1^ (=2000 μE m^−2^ s^−1^). A photoperiod of 12 *h*:12 *h* (light:dark) was adopted to simulate the daily variation of the solar illumination, but the hourly variation of its intensity was not taken into account here due to technical limitation. Continuous illumination during daytime may overestimate the real-world biomass productivity by reducing the peak intensity and alleviating the difficulties of cells to adjust the photosynthetic machinery to variations in light intensity. Such overestimation would decrease in the light diluting schemes, which will be discussed in this manuscript, and we leave more precise investigation of the effect of such hourly intensity variation as a future work. The illumination of 0.6 *sun* with this photoperiod corresponds to 7.2 kWh m^−2^ day^−1^. The illumination area of each bioreactor was confined by apertures and the sides of the reactors were covered by metal (aluminium foil or a stainless steel wall), which prevented overestimation caused by undesirable photon influx from the outside and realized the periodic boundary condition, making it possible for a laboratory-scale reactor to simulate a large system. All the bioreactors were entirely covered by the transparent covers with the same transparent material (i.e. polycarbonate) regardless of their geometry, in order to focus on their geometrical effects rather than material characteristics such as UV-cutting effect. The validity of our system design and corresponding scalability of the system will be demonstrated in our experimental results later.

### Optical study of microalgal photosynthesis

**Figure 1:**
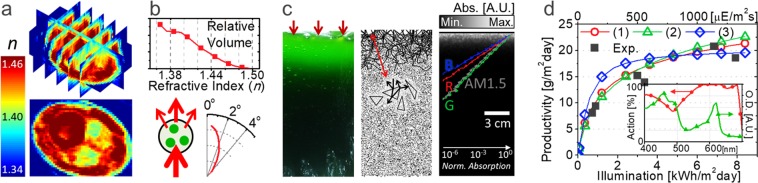
Optical characteristics of microalgal cells. (**a**) 3D profile of the refractive index (*n*) of a *Chlorella vulgaris* cell. (**b**) Volume distribution of the refractive index in the *Chlorella vulgaris* cell (above) and the calculated angular distribution of light scattered by a single cell. (**c**) Picture of a top-illuminated bioreactor (first), ray-traced image of custom-made optical simulation (second), and simulated light absorption (in log scale) with depth under red (680 nm), green (530 nm), blue (440 nm), and solar (AM 1.5 G) illumination (third, the background image is for the AM 1.5 G absorption profile on a linear scale). (**d**) Experimentally measured (black) or modelled (red, green, and blue for the models based on Eqs 1–3, respectively) areal biomass productivity for various illumination (inset: action spectrum used for the modelling and optical density (O.D.) measured for *Chlorella vulgaris*).

Previous optical studies have viewed photosynthesis in a bioreactor as a macroscopic phenomenon^47–56^; therefore, the microscopic optical characteristics of the cells inside the reactor have been rarely studied, and their importance has been scarcely noticed. Here, optical diffraction tomography^57,58^ was used to investigate the biochemical and morphological properties of microalgal cells. Figures 1a and S1 illustrate the 3D refractive index distribution of individual microalgal cells measured at the wavelength of 532 nm. The measured 3D refractive index tomograms of microalgal cells clearly show that individual cells are composed of highly inhomogeneous refractive index distributions and also have refractive indices of 1.36–1.46, which are higher than that of surrounding media (water, *n* = 1.33). Thus, significant amounts of light scattering events can be expected, particularly when there exists large number of microalgal cells. This phenomenon can be quantitatively analysed using the finite-difference time-domain (FDTD) method based on the tomogram (refer to supplementary information (SI)) in Fig. 1b. Such light scattering, although nominal (<2°) with a single cell, becomes substantial with a number density of 10^7^–10^8^ cells mL^−1^ (refer to SI (Fig. S2)).

Light absorption and scattering of the cells result in the uneven distribution of the light energy inside a bioreactor, as shown in the first image of Fig. 1c. As photons are blocked by the cells near the water’s surface, light energy is mostly concentrated within a few centimetres, as is photosynthetic activity; consequently, a negligible degree of photosynthesis occurs in the remainder of the cultivation volume.

Monte Carlo Method can be adopted to investigate and visualize such optical properties^59–61^. To mimic the macroscopic behaviour of microalgae, we designed triangular bubbles and used them as cells for a custom-made optical simulation, as shown in the second image (refer to SI (Fig. S3)). The calculated absorption profiles for red (680 nm), green (530 nm), blue (440 nm), and solar (AM 1.5G) illuminations are shown in the third image of Fig. 1c. Green photons and AM 1.5 G light reach deeper than blue and red photons, which have relatively high extinction coefficients. Such optical characteristics result in a sub-linear productivity with respect to illumination, as shown in Fig. 1d; consequently, the corresponding photosynthesis efficiency (PE) becomes low under increased illumination. The black dots in Fig. 1d represent the experimentally achieved biomass productivity of microalgae (*Chlorella vulgaris*) under various artificial solar illuminations (AM 1.5G).

Modelling of microscopic responses of microalgae will deepen our understanding of their photosynthetic mechanism and the resulting macroscopic behaviours of the photosynthetic system. In previous related studies^47–56^, the PR is mostly represented by a single fitting equation with a sole parameter of total illumination, which ignores the aforementioned optical energy distribution. In the present study, the microscopic PR profile is newly expressed as a function of (*x*, *z*) position modifying the macroscopic models previously proposed. Then, the macroscopic biomass productivity can be obtained by integrating the spatial PR for a whole system. Figure 1d compares such integrated biomass productivities using 3 different models with our experimental results, where the model 1^49,50,56^, 2^47,48^ and 3^54^ are based on

1 $$\begin{array}{rcl}PR(x,\,z) & = & {C}_{{\rm{volume}}}\times {R}_{{\rm{\max }}}\times \,\tanh ((\int {A}_{{\rm{action}}}(\lambda )\times \#Ph.\,(\lambda ,\,x,\,z){\rm{d}}\lambda )\\  &  & /({C}_{{\rm{volume}}}\times {R}_{{\rm{\max }}})\phantom{\int }\,),\end{array}$$

2 $$\begin{array}{rcl}PR(x,\,z) & = & {C}_{{\rm{volume}}}\times {R}_{\max }\times \int {A}_{{\rm{action}}}(\lambda )\times \#Ph.(\lambda ,x,z){\rm{d}}\lambda \\  &  & /({I}_{half}+\int {A}_{action}(\lambda )\times \#Ph.(\lambda ,\,x,\,z){\rm{d}}\lambda ),\end{array}$$

3 $$\begin{array}{rcl}PR(x,\,z) & = & {C}_{{\rm{volume}}}\times {R}_{{\rm{\max }}}\times 2\times {I}_{{\rm{\max }}}\int {A}_{{\rm{action}}}(\lambda )\times \#Ph.(\lambda ,\,x,\,z){\rm{d}}\lambda \\  &  & /({{I}_{max}}^{2}+{(\int {A}_{action}(\lambda )\times \#Ph.(\lambda ,x,z){\rm{d}}{\lambda })}^{2}),\end{array}$$

respectively, where *C*_volume_ is the cell biomass concentration, assumed to be 1.4 g L^−1^, which is the average saturated biomass concentration in our experiments, and *#Ph*.(*λ, x, z*) is the number rate of absorbed photons per volume at a given wavelength and position. *R*_max_ is a constant for the effective maximum PR per weight, influenced by several biological or environmental parameters (species, nutrient, aeration, etc.). *A*_action_(*λ*) is an absorbed-light action spectrum defined as the ratio of photons used for photosynthesis to the total photon absorption at a given wavelength; our chosen species *Chlorella vulgaris* is assumed to have the same spectrum as *Chlorella pyrenoidosa* from previous reports, as shown in the inset of Fig. 1d^62,63^. *A*_action_(*λ*) is relatively low under blue light and becomes ~84**%** for the entire visible light spectrum. Because 48 photons in total are consumed to produce one glucose molecule (29.8 eV) during the photosynthesis process, at least 57 visible photons should be absorbed per 29.8 eV bioenergy produced under low illumination (i.e., Δ*PR(x, z)/Δ#Ph.(x, z)*|_*#Ph*.→0_ = 29.8 eV/57 *photons* = 8.36 × 10^−20^ J/*photon*). This value is the same as that experimentally predicted by others^64,65^. *I*_half_ in Eq. 2 indicates the absorbed active photon densities (=∫ *A*_action_(*λ*) × *#Ph*.(*λ, x, z*) d*λ*) for *PR* reaching the half maximum value, and *I*_max_ in Eq. 3 indicates that for the maximum value. Here, as a boundary condition, we assumed that 57 visible incident photons (i.e. ∫ *A*_action_(*λ*) × *#Ph*.(*λ*) d*λ = *48) are completely consumed to produce one glucose molecule under the extremely low illumination; then, *I*_half_ and *I*_max_ become dependent variables equal to *C*_volume_ × *R*_max_ × 48/(29.8 eV) and 2 × *C*_volume_ × *R*_max_ × 48/(29.8 eV), respectively, and *R*_max_ is the only parameter to be fitted. Figure 1d reveals that the experimental results for flat bioreactors under various illuminations are well matched with our models assuming *R*_max_ = 0.30 W g^−1^ for (1) and *R*_max_ = 0.40 W g^−1^ for (2) and (3). It should be noted that we succeeded in fitting our models to the experimental results even without the terms for photoinhibition and weight loss from respiration, which appear in many previous models and should be considered important. The exclusion of those terms made our model very simple, minimizing the unknown variables; and it was validated by the facts that (i) all our experiments were based on closed photobioreactors with plastic covers absorbing harmful ultraviolet light; and (ii) *R*_max_ fitted to the experimental results can roughly reflect the effect of respiration loss (refer to SI for details). While all the models well represent the optical saturation property of photosynthesis, we chose Eq. 1 for calculating the spatial photosynthesis rates in the rest of this manuscript.

**Figure 2:**
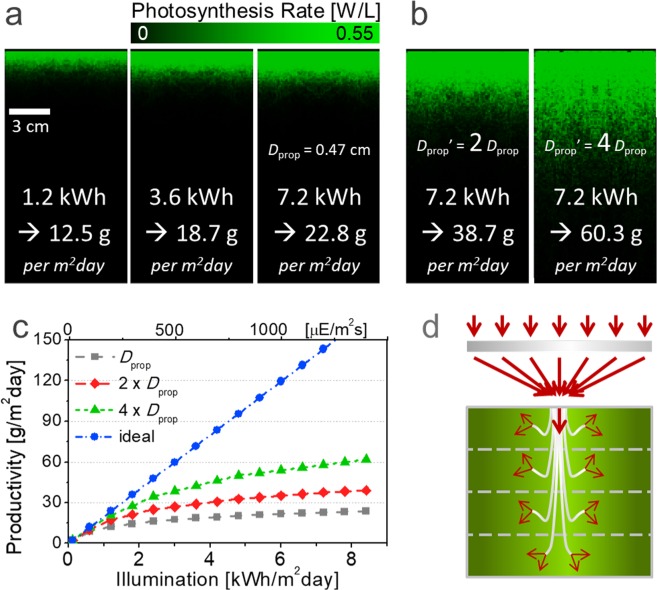
Optical study of microalgal cultivation system. (**a**) Simulated photosynthesis rate profiles and expected biomass productivities under 0.1 *sun* (first), 0.3 *sun* (second), and 0.6 *sun* (third) illuminations. (**b**) Simulated photosynthesis rate profiles under 0.6 *sun* with an increased light propagation of 2*D*_prop_ and 4*D*_prop_. (**c**) Expected biomass productivities of the systems in (**a**,**b**) and the ideal reactor under various illuminations. (**d**) Concept of cultivation building with multiple stacks.

Figure 2a shows the volumetric PR (W L^−1^) inside the top-illuminated flat cultivator based on our new system. A biomass productivity of 12.5 g m^−2^ day^−1^ is estimated under 1.2 kWh m^−2^ day^−1^, which corresponds to 12 hours of 0.1 *sun* (visible photons of 200 μmol m^−2^ s^−1^) illumination, which is a quantitatively similar light intensity to typical light-emitting diodes (LEDs). A combustion heat of 4.2 kcal g^−1^ is obtained from the experiments. Under this condition, the photosynthetic efficiency (PE) of biomass energy is found to be 5.1%. Under such low illumination, the number of photons works as the most deterministic factor of photosynthesis, and the total productivity almost proportionally increases along with the illumination, as shown in Fig. 1d. Under an illumination of 3.6 kWh m^−2^ day^−1^ (12 hours of 0.3 *sun*), which corresponds to the annual average outdoor condition of South Korea, the biomass productivity is increased to 18.7 g m^−2^ day^−1^. Compared with the 1.2 kWh m^−2^ day^−1^ illumination, as shown in Fig. 2a, photosynthesis occurs more deeply and more intensively; the 50% increase in total productivity, by contrast, is much less than a 3-fold illumination boost; therefore, the PE is reduced to 2.5%. This reduced efficiency occurs because the photon flux is saturated near the surface. Likewise, under a higher illumination of 7.2 kWh m^−2^ day^−1^ (12 hours of 0.6 *sun*), which corresponds to the daily illumination of the world’s hottest regions (such as the Sahara Desert and the Andes Mountains), the biomass productivity increases to 22.8 g m^−2^ day^−1^; however, the PE drops to 1.5%, as the excess photons are not efficiently utilized. It should be noted that the areal productivity of 22.8 g m^−2^ day^−1^ corresponds to the volume productivity (=areal productivity/reactor depth) of 0.46, 0.23, and 0.08 g L^−1^ day^−1^ for the reactors with a depth of 5, 10, and 30 cm, respectively. Such optical inefficiency, unless resolved, thwarts the installation of photosynthetic systems in highly illuminated places.

Effective alleviation of the optical inefficiency, particularly under high illumination, is therefore a key to achieving an economically feasible level of biomass productivity. For a biomass concentration of 1.4 g L^−1^, which was assumed here, a light propagation depth (*D*_prop_) of 1/α (α: 2.1 cm^−1^) is only 0.47 cm. As shown in Fig. 2b, hypothetically assuming that photons can propagate twice and 4 times deeper with the same cell concentration, the expected biomass productivity would be increased to 38.7 g m^−2^ day^−1^ and 60.3 g m^−2^ day^−1^, respectively, even under the same illumination. Figure 2c presents the expected biomass productivity of those virtual conditions as a function of illumination. The productivity difference increases under higher illumination, whereas the productivity is nearly saturated in the original system. In particular, for an ideal system, which sufficiently dilutes incident light, a PE of 9.7% can be achieved for all illumination conditions, and a biomass productivity of ~140 g m^−2^ day^−1^ is achievable under high illuminations of >7.2 kWh m^−2^ day^−1^. While the genetic engineering approach must be continuously sought to increase *D*_prop_ by reducing the absorption cross-section of each cell^64^, optical approaches with system design would possibly offer an equally effective and/or synergistically better means to it. A futuristic, potentially ideal cultivation system can be constructed as a form of *cultivation building* by inserting light guides into the deep or multiply stacked bioreactor, as schematically illustrated in Fig. 2d^16–22^. In the cultivation building, light energy can be evenly distributed through the guides such as optical fibers; however, this concept has been rarely realized before and there are inevitable limitations associated with it, such as structural complexity, high-cost installation of optical components, and high-cost operation of light tracking, which must be accompanied to fix the focus of concentrated light to the light guide according to the solar movement. As a more practical light dilution approach, planar or tubular type photobioreactors are widely used^23–33^, but performance is inferior to the ones in Fig. 2c, as discussed in the next section.

### Practical configuration for boosting productivity

**Figure 3:**
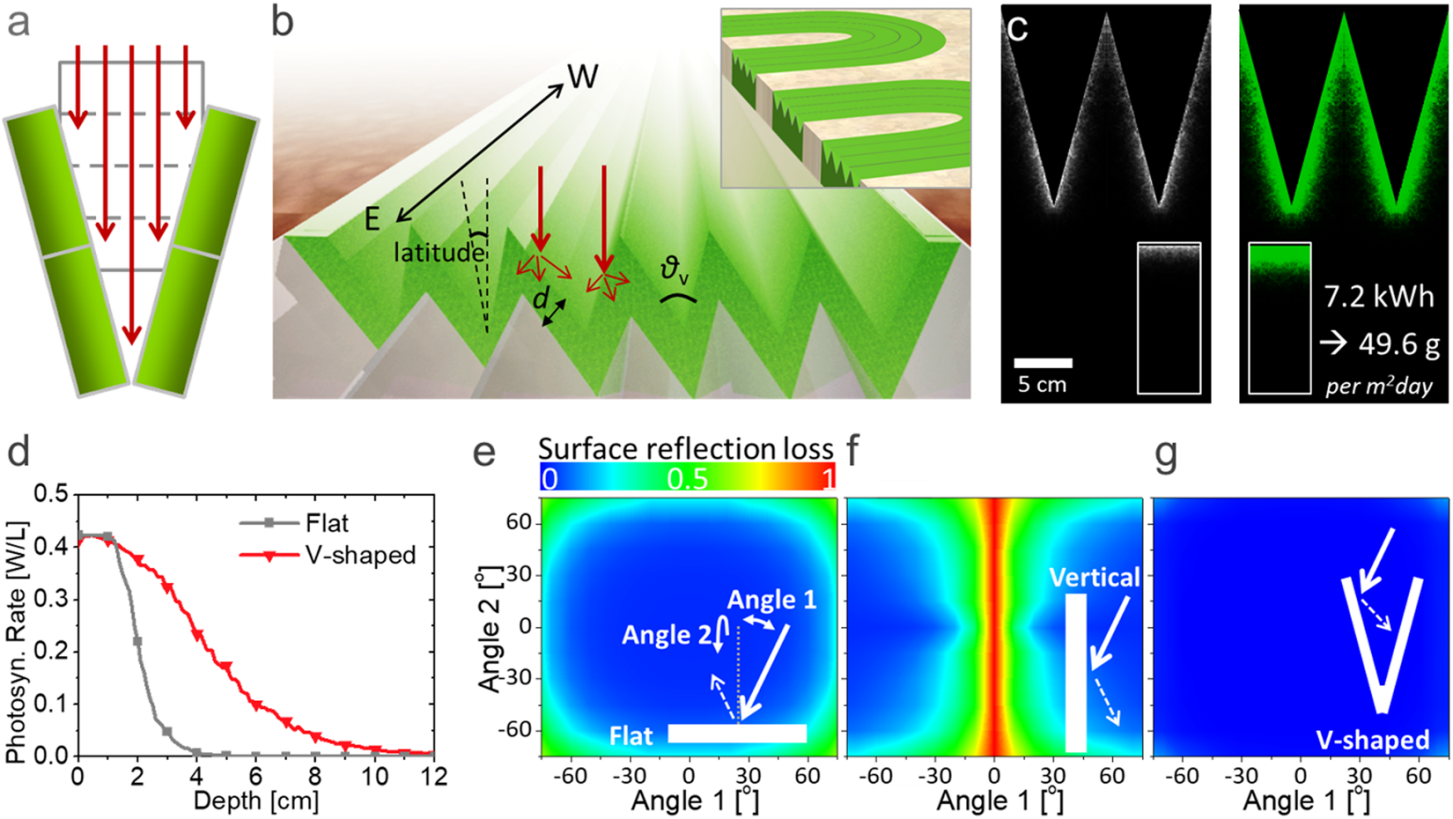
Optical modelling for V-shaped microalgal cultivation. (**a**) Cultivation building modified to the V shape. (**b**) V-shaped cultivation array system. (inset: a raceway pond with a V-shaped cover) (**c**) Simulated absorption (left) and photosynthetic rate (right) profiles of the V-shaped system (inset: those for flat systems) (**d**) Average photosynthesis rate as a function of depth. (**e–g**) Calculated Fresnel reflection loss on the surface of (**e**) the flat system, (**f**) the vertical plate, and (**g**) the V-shaped system for an incident angle change parallel (angle 1) or perpendicular (angle 2) to the cross-section plane.

To realize the light dilution in an easily implementable fashion, we modified the configuration of shown in Fig. 2d in such a way that each stack is inclined, as shown in Fig. 3a. As a result, the incident sunlight is evenly distributed among the stacks without requiring high-cost optical components. This scheme converges to a V-shaped array cultivator^25–27^, as presented in Fig. 3b, of which concept is simple and easy, but has been less spotlighted and rarely studied scientifically. The inclined surfaces of the V-shaped system receive sunlight over a larger area, and the light intensity is diluted by sin(*θ*_v_/2), where *θ*_v_ is the vertex angle. Hence, the V-shaped configuration is equivalent to the system shown in Fig. 2b with *D*_prop_’=*D*_prop_/sin(*θ*_v_/2) or the cultivation structure in Fig. 2d with 1/sin(*θ*_v_/2) stacks. If the system is aligned along the east-to-west line and properly tilted according to the latitude of the installed location, the incident angle of sunlight at noon can be restricted within only ±23.5° from the normal line for an entire year^66–69^. The system can be alternatively realized by implementing a V-shaped cover on top of a raceway pond (inset of Fig. 3b), which makes the V-shaped system economically more viable compared to previous light propagation enhancement systems that are mostly restricted to photobioreactor types or use high-cost optical components^70–72^. The V-shaped scheme is very scalable as well (refer to SI (Fig. S4)); what is better, the material cost does not depend on the depth of the pond. The cover can be made of various transparent materials and it would increase the installation cost. However, the cost would be compensated for by an enhanced algae productivity of more than 100%. The techno-economic analysis shown in SI (Fig. S6) reveals the V-shaped cover has a potential for reducing the microalgal biomass selling price from ~US$540 ton^−1^ to ~US$280 ton^−1^ in an open pond with the improved productivity. Moreover, by blocking the interface with air, the implementation of the cover would also be beneficial for reducing the operation cost by minimizing water evaporation, which accounts for up to 67% of the total water use, and CO_2_ consumption, which mostly escapes to the air due to a low water solubility and accounts for 15–20% of the total price of the dry biomass^15^.

Our optical system modelling of photosynthesis enables the prediction of the photosynthetic performance for non-planar-type cultivation. Figure 3c shows a simulated optical energy distribution in a V-shaped system with *θ*_v_ = 30°, which is equivalent to the cultivation structure comprising 3.9 stacks. Compared to the flat system (inset), incident light reaches much deeper and the integrated photosynthesis depth profile becomes more uniform, as shown in Fig. 3d. The total biomass productivity is expected to be 49.6 g m^−2^ day^−1^, which is 118% higher than that of the flat system and between those for *D*_prop_’ = 2 *D*_prop_ and *D*_prop_’ = 4 *D*_prop_ (Fig. 2b,c).

In fact, the V-shaped configuration was proven to be one of the most efficient light-trapping schemes in a PV study^67,73,74^. Figure 3e–g present the Fresnel reflection loss on the air/glass/water interfaces of a ground-type reactor, a vertical plate reactor, and a V-shaped reactor, respectively. As the Fresnel reflection increases as the incident angle increases, the reflection loss is considerable, especially in the vertical plate; the loss becomes highest at small angles 1 and 2 (inset of Fig. 3e), which correspond to summer or noon with high illumination. Therefore, a large amount of loss follows. However, such losses can be almost fully avoided by the V-shaped geometry because the photons escaping from one side can enter the opposite side.

### Comparative study of various configurations

**Figure 4:**
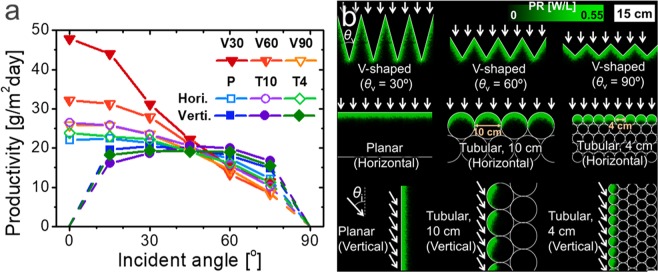
Comparative study for various cultivation systems. (**a**) Calculated areal biomass productivities of V-shaped cultivators (*θ*_v_ = 30°, 60°, and 90°, denoted by V30, V60, and V90, respectively) and planar (P) or tubular (T10 for diameter of 10 cm and T4 for diameter of 4 cm) cultivators horizontally or vertically installed on the ground as a function of the incident angle on the cross-sectional plane. (**b**) Photosynthesis rate (PR) profiles inside the systems in (**a**) with the same scale bar, at the incident angle of 0° for the horizontally installed systems and 30° for the vertically installed systems as represented by the arrows.

While there have been numerous approaches previously proposed for light dilution, the system modelling designed here makes it possible to quantitatively compare the different geometry and optimize the best architecture. To make a general comparison, we classified and simplified the previously proposed configurations into a few 2D cross-sectional geometries including (i) horizontally placed planar geometry (e.g. open-pond, top-illuminated bioreactors with a flat surface, etc.); (ii) vertically placed planar geometry obliquely receiving sunlight (e.g. vertical plat panel, vertical cylinder, etc.); (iii) horizontally placed circles array (e.g. horizontal tubular bioreactors, etc.); and (iv) vertically placed circles array (e.g. fence tubular, helical tubular, spiral bioreactors, etc.), varying vertex angle of V-shape or radius of circles as shown in Fig. 4. The illumination was roughly assumed to be 7.2 cos(*θ*_i_) kW m^−2^ day^−1^ where *θ*_i_ indicates the incident angle on the cross-sectional plane of each system shown in Fig. 4b. For the vertical systems, the geometrical fill factor was assumed to be dense enough to absorb the full illumination without optically dead area on the ground.

The V-shaped systems with vertex angles of 30° and 60° are shown to much outperform the other systems near the normal incident angle (*θ*_i_ = 0°), while that of 90° is similar to the one for tubular reactor with a diameter of 10 cm. The biomass productivity at the normal angle is shown to increase as the vertex angle decreases and the factor of light dilution (=1/sin(*θ*_v_/2)) increases. On the other hand, for the larger incident angle (*θ*_i_ > 1/sin(*θ*_v_/2), the incident light impinges on only one side of the V-shaped bioreactor, and the illuminated area decreases as the angle increases further. As a result, the light dilution effect is reduced and the productivity drops rapidly. Concentrating the light dilution effect near the normal incident angle is an effective strategy since the diluting effect is especially critical for the high illumination with the high altitude of the sun. Aligning the bioreactor can further minimize the cross-sectional angle variation as described in Fig. 3b; the annual variation of the incident angle at noon can be restricted to ±23.5°.

The horizontally installed planar bioreactor has shown the minimum variation of the productivity along the variation of the incident angle, since the factor of light dilution is always 1 regardless of the incident angle. On the other hand, the effect of light dilution was shown to be not significant for the vertically installed planar reactor for all angles, despite a dilution ratio of 1/sin(*θ*_i_). In the vertical systems, the angle between the light and surface becomes shallow as the incident angle decreases, and the corresponding Fresnel reflection increases, as discussed in Fig. 3f; the productivity becomes particularly very low near the normal incident angle. Moreover, the increased cost for high density installation to prevent the optical loss also plays a negative impact on their economic viability.

Tubular reactors may compensate for such limitations of the planar reactors. The circular surface itself has a light dilution effect in manner that increases the surface area by π/2 and reduces the Fresnel reflection loss by geometrically guiding the reflected light. Hence, biomass productivity of the horizontally installed tubular system can be higher than that for the planar system at some angles. For the diameter of 4 cm that is comparable to the optical propagation distance in dimension, the geometrical effect is relatively low and the performance is close to that for planar system.

It is this reason that we determined the V-shaped configuration as the most efficient system among the examined candidates, at least in the optical point of view. In addition to this, the V-shaped system is distinctively advantageous in that implementation is easily done by simply placing plastic cover on top of an open-pond, while vertical and/or tubular bioreactors are inherently costly. More precise comparison of non-optical efficiencies such as shaking and aeration, possibly influenced by geometry, remains as a future work.

### Experimental demonstration

**Figure 5:**
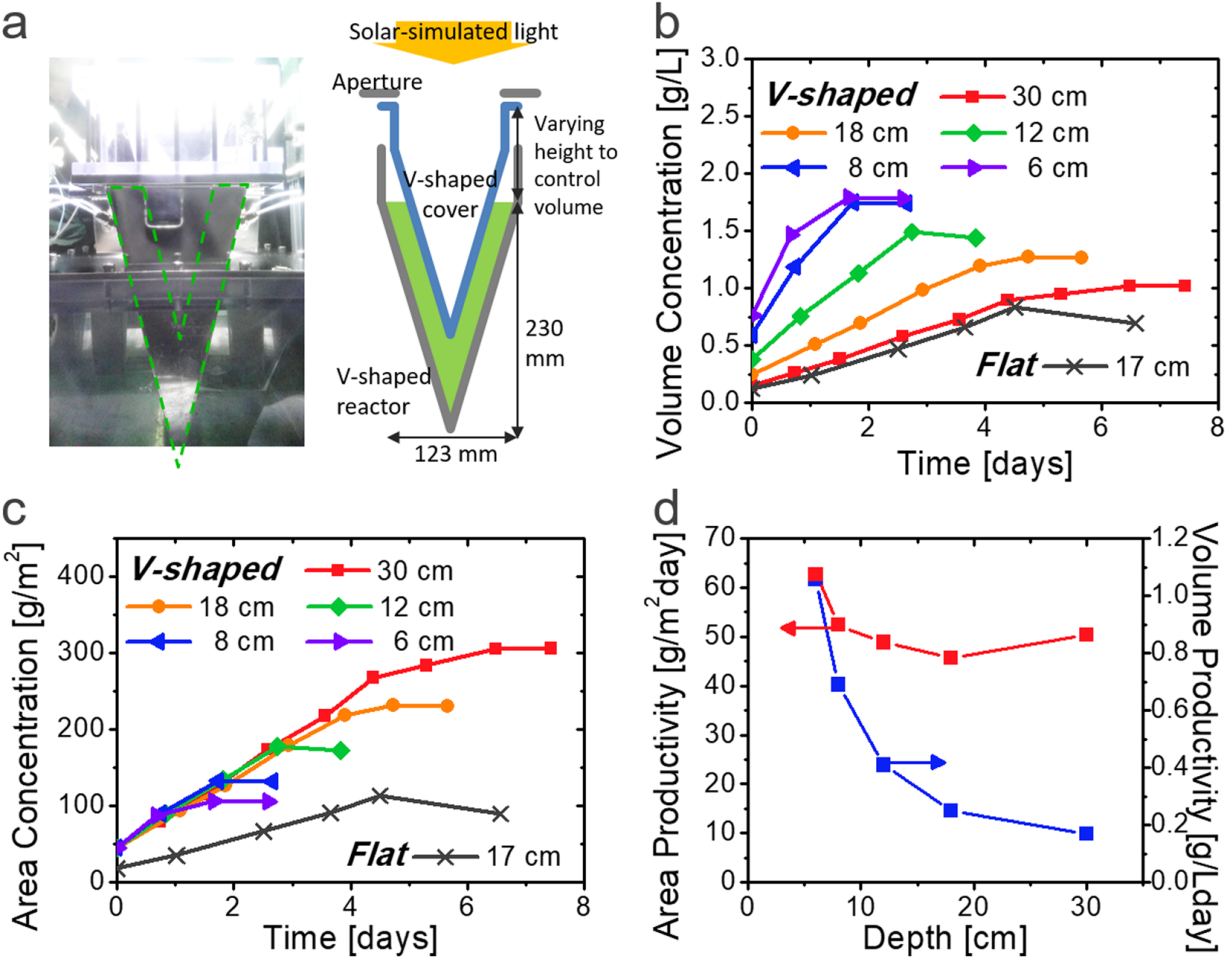
Experimental demonstration of V-shaped microalgal cultivation. (**a**) Photograph and diagram of the V-shaped bioreactors used in the experiment. (**b**,**c**) Measured (**b**) volume and (**c**) area concentration of microalgal cells during growth in the flat and V-shaped systems with various depths. (**d**) Volume and area productivities of microalgal biomass in the V-shaped systems with various depths.

Experimentally, we constructed a V-shaped physical bioreactor with *θ*_v_ = 30°, as shown in Figs 5a and S7. Under 0.60 *sun* and 12 *h*:12 *h* (*light:dark*) conditions, the growth profiles with different system depths are presented in Fig. 5b–d. Although the volume concentration is saturated more quickly in the shallow system than in the deep system, the total growth rate per given illumination area is shown to be almost similar, 52.0 g m^−2^ day^−1^ on average, which is 2.5 times higher than that of the flat bioreactor (20.7 g m^−2^ day^−1^). The PE was also improved from 1.40% to 3.52%, in accordance with the our modelling results of the system (Figs 2c and 3c). The consistent productivity even with a small-volume V-shaped bioreactor can be attributed to the light-trapping effect, which makes it possible to compensate for the short optical path length of shallow depth. The final cell concentration tends to be higher for systems with small volumes, yielding a higher economic viability by reducing the cost for product drying, water supply, and nutrient preparation. The enhanced microalgal growth in a V-shaped bioreactor was consistently demonstrated in several repeated experiments with semi-continuous cultivation, varied environmental conditions, and a large-volume cultivation system as shown in SI (Fig. S5).

The main purpose of this research indeed lies not in proposing a single outstanding scheme *per se*, though we believe ours is such a one indeed, but rather in applying a quantitative optical engineering approach to microalgae research, which has rarely been done in a systematic way. The V-shaped configuration is a mere example proposed through the optical study by means of comparing and integrating all types of previous attempts. Beyond the academic studies, real-world scale demonstration of the proposed scheme would necessarily be a future step for commercializing microalgae and contributing to recovering global carbon balance. The practical issues such as water circulation affected by V-shaped cover, off-gassing of generated oxygen, and lifetime of plastic components, which have not been deeply investigated here, could be raised for scaling up the schemes. Moreover, analysis of lipid contents of biomass, critical for biofuel extraction, as a function of light input and integration with genetic engineering would bring further benefits for the economic viability of microalgal biomass.

## Materials and Methods

### Cultivation environment

*Chlorella vulgaris* from the University of Texas (UTEX-265) was cultivated in a BG-11 medium, which contained 1.5 g L^−1^ of NaNO_3_, 0.075 g L^−1^ of MgSO_4_·7H_2_O, 0.020 g L^−1^ of Na_2_CO_3_, 0.036 g L^−1^ of CaCl_2_·2H_2_O, 0.006 g L^−1^ of citric acid, 0.001 g L^−1^ of disodium ethylenediaminetetraacetate (Na_2_EDTA), 0.040 g L^−1^ of K_2_HPO_4_, 0.006 g L^−1^ of ammonium ferric citrate, and 0.1% v/v of trace element solution (Oilgae) in deionized (DI) water^42,45,75^. The environmental conditions were maintained for all reactors with a temperature of 27 °C, pH of 7.0, and aeration (CO_2_ 5% v/v, 0.3-1.0 vvm). The V-shaped reactors had various volumes of 684 mL, 394 mL, 258 mL, 164 mL, and 128 mL and a fixed illumination area of 21.6 cm^2^. The dual-energy generator had a volume of 500 mL and an area of 45 cm^2^. Stainless steel reactors with polycarbonate covers were shaken at >80 rpm during the experiment. Visible light transmission of the reactor cover was found to be 77.1%, which was considered for the fitting in Fig. 1d. Autoclaved DI water was injected periodically to compensate for water evaporation.

### Analysis

The optical densities (OD = −log_10_ *Transmission*) of diluted samples (0.5 mL solution + 2 mL DI water) at 680 nm wavelength were measured using a UV-vis spectrometer (UV-3600 Plus, Shimadzu, Japan) every day and converted to the dry cell weight concentration based on calibration data. The calibration was performed one day after the growth phase ended. The PE was calculated as [(biomass productivity per area) × (heat value per biomass)/(illumination per area)], where the heat value (4.2 kcal g^−1^) was measured using a calorimeter.

### Statistical analysis

Due to the large environmental dependency of microalgal cultivation, we concluded that the representation of the biomass productivities with a single average value and standard deviation may not be trustworthy enough and easily distort the conclusion. Therefore, to avoid the possible misinterpretation and obtain the confidence of the results, the experimental results for semi-continuous cultivation, various environmental conditions, and larger volume reactors are shown with the raw growth curves in SI (Fig. S5).

## Conclusion

In this study, the optical inefficiency of microalgal photosynthesis was revealed through advanced system modelling based on both microscopic 3D tomography and macroscopic photosynthesis profile. While the cultivation of microalgae has been mostly studied in the field of bioengineering, we have shown that optical study is a key to further boost the biomass productivity to >100 g m^−2^ day^−1^ by making photons penetrate longer distance into the bioreactor. We proposed a V-shaped cultivation as a practical scheme for trapping and diluting sunlight. Our modelling work verified that the V-shaped cultivation can achieve the nearly doubled biomass productivity within the incident angle variation of ±23.5°, compared to the previously proposed photobioreactors of vertically or horizontally installed planar or tubular configurations. Experimentally, we verified that the V-shaped configuration can enhance the biomass productivity from 20.7 g m^−2^ day^−1^ to 52.0 g m^−2^ day^−1^ under 7.2 kWh m^−2^ day^−1^ with an outdoor-simulating environment and such areal productivity was shown to be consistent regardless of the cultivation volume, leading higher volume productivity in shallow reactors.

## Supplementary information

Supplementary Information

## Data Availability

The datasets generated and/or analysed during the current study are available from the corresponding author upon reasonable request.
