# Supplementary material for: Study of Optical Configurations for Multiple Enhancement of Microalgal Biomass Production
Source: Sci Rep. 2019 Feb 11;9:1723. doi: 10.1038/s41598-018-38118-w (PMC6370833; doi:10.1038/s41598-018-38118-w)
Supplement: Supplementary file 1 — Supplementary Information [file 41598_2018_38118_MOESM1_ESM.pdf]

Supplementary Information

Study of Optical Configurations for Multiple Enhancement of Microalgal Biomass Production

Changsoon Cho, Kibok Nam, Yeong Hwan Seo, Kyoohyun Kim, YongKeun Park, Jong-In Han*, and Jung-Yong Lee*

Optical diffraction tomography

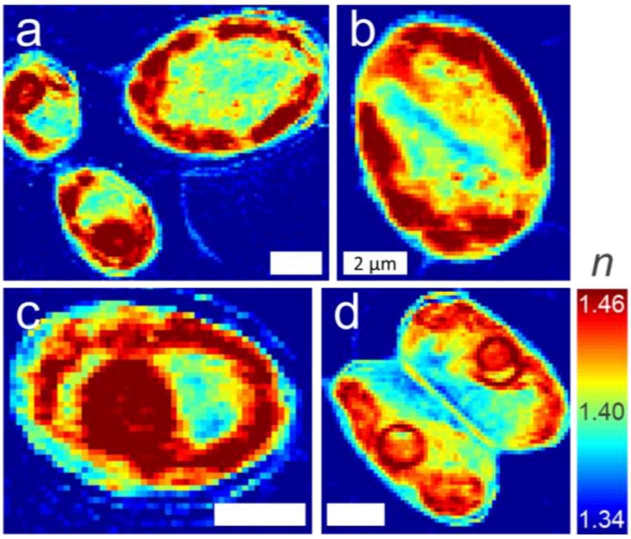

Figure S1. (a-d), 3D holographic images of various *Chlorella vulgaris* cells.

The 3D refractive index distribution of an individual cell was measured by implementing optical diffraction tomography. In the optical diffraction tomography setup, a collimated laser beam (λ = 532 nm, Cobolt AB, Sweden) illuminated the cell at various incident angles by tilting a galvanomirror. The diffracted beam from the sample was collected by a high-NA oil-immersion objective lens (NA = 1.4, 100 $\times$, Olympus, Inc., Japan) and a tube lens. Mach-Zehnder interferometry was employed to interfere with the diffracted beam and a reference beam to generate spatially modulated holograms, which were recorded by a CCD camera. From the recorded holograms, the optical fields of the samples from various incident angles were retrieved, and an ODT algorithm based on the Fourier diffraction theorem reconstructed the 3D refractive index distribution of the sample from the retrieved complex optical fields. Optical diffraction tomography provides label-free images with high spatial resolution: a lateral resolution of 108.6 nm and an axial resolution of 255 nm, which are calculated from the maximum range of Fourier frequency to be achieved by the objective lens. Detailed information on the experimental setup and ODT can be found elsewhere.[1,2]

Acquired images are shown in Figure 1a and S1. For the first image of Figure 1b, the refractive index distribution was obtained from 20 cells.

Light scattering of microalgae cells

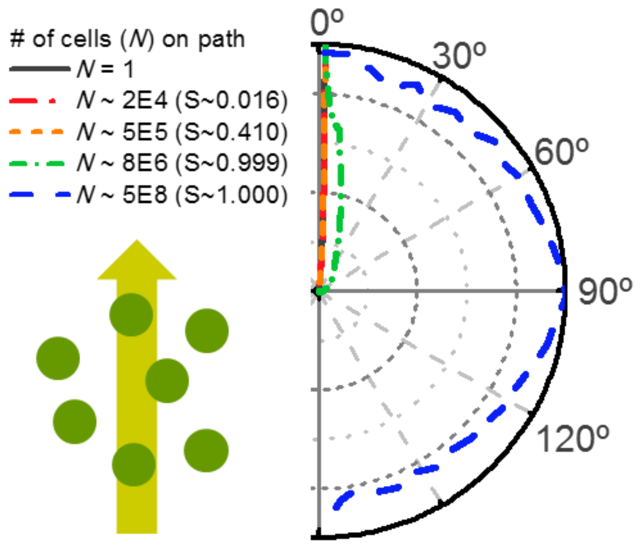

Figure S2. Calculated scattering ratio (S) and normalized angular distribution of scattered light after passing an optical path containing $N = 1$, 2×10^4, 5×10^5, 8×10^6, and 5×10^8 microalgae cells, respectively, with a refractive index profile shown in Figure 1a. Angular distribution is represented in "*per unit projected area*" (i.e. total intensity / sin (θ_{prop})), where Figure 1b is in total intensity.

It is well known that microalgae cells have a considerable amount of light absorption and scattering properties; however, those optical properties have never been quantitatively analyzed before. The single cell analysis with 3D tomography and finite-difference time-domain (FDTD) simulation shown in the second image of Figure 1b would make it possible to study at least the macroscopic behavior of the cells. For FDTD simulations, a commercial program (FDTD Solutions, Lumerical, Inc.) was used with a total-field scattered-field (TFSF) light source to record the angular distribution of the scattered light. The refractive index profile was reconstructed by hexahedrons in the domain with sufficiently small meshes, surrounded by perfectly matched layers (PML) boundaries

Figure S2 shows the calculated distribution of light propagation angle (θ_{prop}) after multiple scattering events at cells inside the reactor. Each scattering profile was calculated by recursively adopting single cell's scattering profiles (Figure 1b). The scattering ratio is shown to become almost unity when $N = 10^6$ and the angular distribution becomes fully randomized when $N = 10^8$. Therefore, light scattering increases as the optical path increases or cell concentration increases in the media. For the modelling

works (Figure 1c), on the other hand, we mimicked such scattering properties using triangular bubbles with a reduced number density of 770 cm^{-2}. The simulation results with this number density have shown to fit the macroscopic light absorption of microalgal solution with biomass concentration of 1.4 g/L.

Respiration effect on photosynthesis model

In the manuscript, Equation 1 has only R_{max} as a fitting parameter and we assumed that all the environmental factors are included in this *experimentally-fitted* model. However, a more precise model would be required when each environmental factor needs to be separately studied. For example, for the net photosynthesis rate separating the respiration loss, Equation 1 can be modified in the following way:

$$PR_{net}(x, z) = C_{volume} \times R'_{max} \times tanh\ ((\int A_{action} \times \#Ph.\ d\lambda) / (C_{volume} \times R'_{max})) - C_{volume} \times R_{respiration} \quad (S1)$$

R'_{max} is the maximum PR per weight excluding the respiration effect. $R_{respiration}$ is the rate of respiration per weight in W g^{-1}, which can be calculated by (4.2 kcal g^{-1}) × (4180 J $kcal^{-1}$) / (86400 s) × (weight ratio of the daily respiration loss to the total weight). According to the previous literatures,[3-6] the last term is typically known to be in the range of 2 – 10%, making $R_{respiration}$ to be 0.004 – 0.02 W g^{-1}, of which precise value should be determined from experiments and may deviate the scope of this study. At the illuminated region with high *#Ph*, the *tanh* term becomes close to one and the respiration term of $C_{volume} \times R_{respiration}$ becomes negligible because $R'_{max} > R_{max} = 0.30$ W g^{-1} >> $R_{respiration}$. On the other hand, at the dark region, the *tanh* term becomes zero and $PR_{net}(x, z)$ becomes negative, reducing the total areal biomass productivity. Therefore, for the fixed cell concentration, total areal productivity decreases as the depth of reactor decreases and dark region increases. Hence, for the fixed R'_{max} and $R_{respiration}$, our R_{max} fitted to the experiment using 17 cm depth is expected to increase or decrease for shallower or deeper reactor, respectively.

Optical Simulation

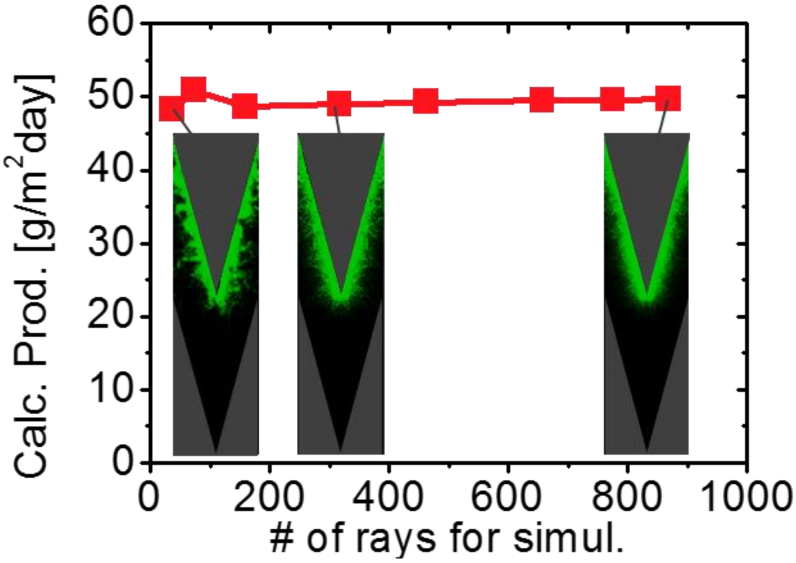

Figure S3. Calculated biomass productivity of the V-shaped cultivation shown in Figure 3c as a function of the number of rays used in the simulation. Inset images represent the photosynthesis profile for the number of 37, 318, and 870, respectively.

The optical simulations were based on a custom-made program calculating the 2D ray-optical propagation of light.[7-9] Since the direct implementation of real 3D microstructures shown in Figure 1a requires a very large memory and a long runtime, we instead represent the optical properties of microalgae cells by means of embedding virtual simple structures with similar light-scattering and -absorbing characteristics. Inside the water medium (n = 1.33), triangular air bubbles (n = 1) were generated and randomly positioned with a size of 100 μm, and they were assumed to absorb a low percentage of the light bouncing on their interfaces and to split the remaining energy into transmitted and reflected rays according to the Fresnel equation. With these conditions, we found that the system with 770 bubbles cm^{-2} on the cross-section of the 2D domain shows the optical property corresponding to the microalgal solution with an average extinction coefficient (α) of 2.1 cm^{-1} in the visible range, appearing at the dry weight concentration of 1.4 g L^{-1}. The reactors were assumed to have a plastic wall (n = 1.49) with a 2 mm thickness and aluminum foil on the rear side for the all simulation works. The simulation regions were assumed to have a periodic boundary condition (PBC).

The energy absorption profile was constructed by dividing the simulation region into 1 mm size meshes and colouring them based on the absorption data obtained by the optical simulation. The local and total energy densities were used as inputs of Equation (1) in the manuscript. The number of the rays per unit structure was determined considering the accuracy and simulation running time. As shown in Figure S3, we found that the deviation of the result becomes <1 % and the photosynthesis profile becomes smooth when the number is larger than 400. We used >800 rays to secure the high accuracy for the simulations in the manuscript.

Scalability

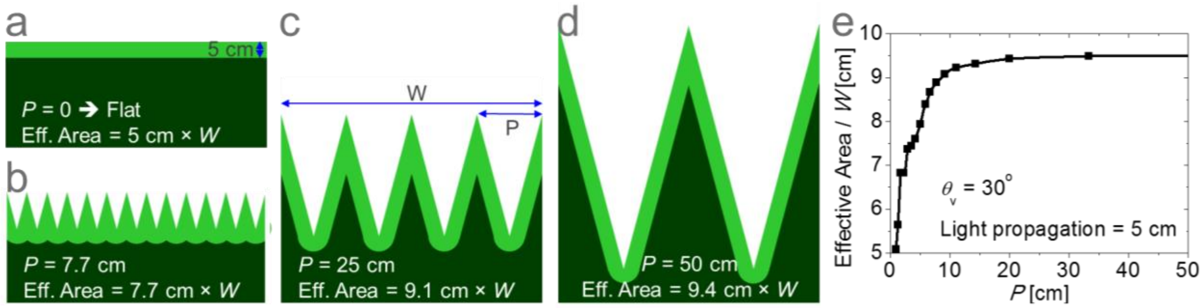

Figure S4. (a-d) The depiction of the area (light green) within 5 cm from the illuminated surface of the V-shaped open pond (θ_v = 30º) with a period (*P*) of (a) 0 cm, (b) 7.7 cm, (c) 25 cm, and (d) 50 cm. (e) Effective area over the system width (*W*) as a function of *P*.

On a large scale, the optical property of the V-shaped system is not influenced by the dimension of the structure. However, when the scale decreases to be comparable to the effective depth that light can penetrate, the effective volume that receives light can be reduced by the overlap between the periodic structures. For example, for a V-shaped structure with θ_v = 30º and period (*P*) of 50 cm, the effective area within 5 cm from the illuminated surface is 9.4 cm × *W* on the cross-sectional plane shown in Figure S4d. By contrast, for the extreme case with *P* → 0 shown in Figure S4a, the effective area (5 cm × *W*) becomes the same as that for the flat configuration. Figure S4b, c and d show the effective area for *P* = 7.7 cm, 25 cm and 50cm, respectively, which are enlarged as *P* is elongated until saturation, as shown in Figure S4e. Therefore, to achieve the sufficient optical effect of a V-shaped configuration with θ_v = 30º, the construction rule can be given that the structure period should be secured to be longer than at least twice the effective light propagation depth.

Indeed, our simulation (49.6 g m^{-2} day^{-1}) and experimental (52.0 g m^{-2} day^{-1}) results of the V-shaped system (θ_v = 30º, *N* = 3.9) with *P* of 8-9 cm are not as high as the one for the theoretical value of 57.9 g m^{-2} day^{-1} for 4 × D_{prop} in Figure 2c. Lower productivity may be partly due to the scalability, the issue discussed above. While the cost for typical optical approaches such as light guiding structures soars as the structural dimension is increased, the material use for the V-shaped cover is only proportionally so

to $1/\sin(\theta_v)$ regardless of the dimension, so the economic viability remains unaffected. The maximum dimension of P would be limited by the depth of pond. For the typical open pond with 30 cm depth, the V-shaped cover with the height of 50 cm (P = 27 cm for $\theta_v = 30^o$) results in the total 55 cm-deep pond with 5 cm space from the bottom to the vertex, remaining the total cultivation volume unchanged and enhancing the effective illuminated volume almost twice.

Reproducibility of the growth curves

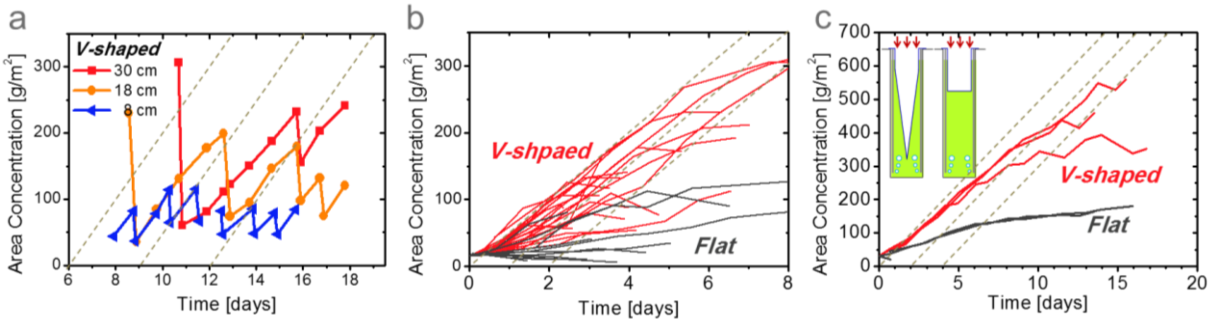

Figure S5. Growth curves of (a) semi-continuous cultivation of V-shaped bioreactors with depths of 30 cm, 18 cm, and 8 cm. (b) V-shaped and flat cultivation with various environmental conditions. (c) Cultivation using large-volume reactors with V-shaped (4.4 L) and flat (5.1 L) surfaces during the growth phase (inset: the shapes of the reactors). Dotted lines represent a slope of 50 g m^{-2}day^{-1}.

The productivities of the V-shaped bioreactors shown in Figure 5c were confirmed by semi-continuous cultivation, as presented in Figure S5a. Irrespective of the volume, the slopes of the growth curves were almost consistently maintained, and an area productivity near 50 g m^{-2}day^{-1} was achieved every cycle. As the saturation point is rapidly reached in the bioreactor with small volume, the harvest period is shown to become shorter than other bioreactors.

Figure S5b shows the collection of growth curves of V-shaped and flat bioreactors with various environmental conditions during our experiments for optimizing aeration, shaking, temperature, volume and material for the reactor, while the illumination power was fixed to 7.2 kWh m^{-2}day^{-1}. The biomass productivities of V-shaped bioreactors are shown to be relatively more consistent and higher than those for flat bioreactors.

Whereas the growth curves were obtained with small-volume reactors (< 700 mL) for Figure 5c and S5a-b, Figure S5c shows the curves for the growth phase in large-volume reactors of 4.4 L and 5.1 L for the V-shaped and flat configurations, respectively. For those reactors, the illumination area was 57 cm^2 and the effective depth was greater than 70 cm. The aeration was reduced from 1 vvm to 0.4 vvm as the path length of the bubbles inside the water medium increased. Both of the reactors have a rectangular shape and were covered by a V-shaped or flat cover, as depicted in the inset of

Figure S5c. A vertex angle of 15º was used for the V-shaped cover to manage the increased depth. As shown in the graphs, the slopes of the growth curves were clearly enhanced by adopting the V-shaped cover. The experiment was repeated 3 times, and the average productivity was shown to be 44.0 g $m^{-2}day^{-1}$ and 13.6 g $m^{-2}day^{-1}$ for the V-shaped and flat reactors, respectively, which are slightly lower than those for a smaller volume, possibly due to the increased dark volume, where only respiration occurs without photosynthesis.

Economic issues related to the proposed schemes

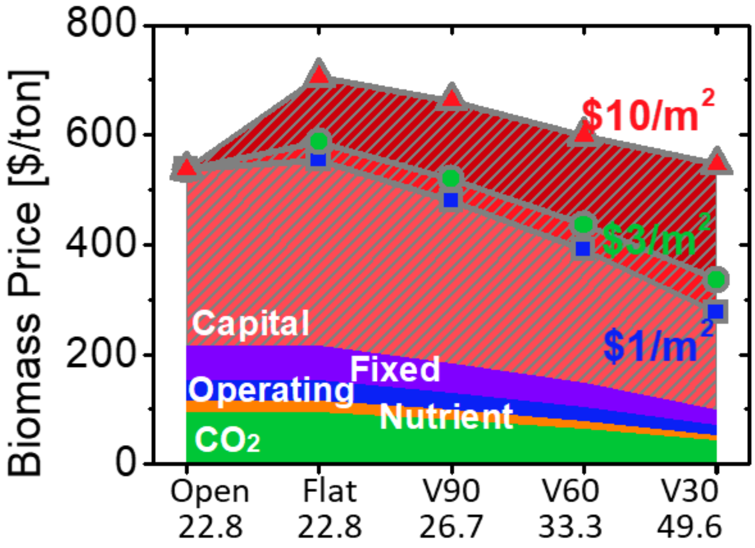

Figure S6. The estimated selling price of microalgal ash-free-dry weight biomass for cultivation ponds without cover, with a flat cover, and with V-shaped covers having a vertex angle of 90°, 60°, and 30°, respectively. The numbers below the name of each system indicate the computed biomass productivity in g m^{-2} day^{-1} under the illumination of 7.2 kWh m^{-2} day^{-1}. Green, orange, blue, and violet regions indicate the cost for CO_2 supply, nutrient supply, other operating cost (power and chilled water utility), fixed operating cost (labor, etc.), and capital-related cost, respectively. The capital-related cost varies depending on the material price of transparent cover and the values for $1, $3, and $10 m^{-2} are plotted.

In Figure S6, the techno-economic analysis model was established on the basis of a technical report[10] previously presented. From the reported data for a 10-acre sized raceway pond, we assumed the annual costs for CO_2 consumption of $0.77 m^{-2} yr^{-1}, ammonia and diammonium phosphate consumption of $0.18 m^{-2} yr^{-1}, electricity consumption of $0.27 m^{-2} yr^{-1}, chilled water utility of $0.03 m^{-2} yr^{-1}, fixed operating costs (for labors) of $0.52 m^{-2} yr^{-1}, and no cost for makeup water, assumed to be supplied from a nearby groundwater resource. The annual cost for capital investment was calculated assuming the added direct and indirect costs of 39 % (of total investment), internal rate of return (after-tax) of 10 %, equity percent of total investment of 40 %, loan rate of 8.0 % (10 years),

and the calculated capital charge factor of 0.137. Then, the total capital investment of \$390,200,000 per 5,000 acres pond was converted to the annual cost of \$2.64 m^{-2} yr^{-1}, while the capitals for raceway ponds, inoculum ponds, CO_2 delivery, water circulation (and makeup water delivery), dewatering, and storage take 40.3 %, 4.2 %, 4.9 %, 11.4 %, and 39.4 % of it, respectively. Then, the total selling price of microalgal ash-free-dry weight biomass becomes \$536 ton^{-1} for a pond with the productivity of 22.8 g m^{-2} day^{-1}.

If the V-shaped cover is placed on the open pond, more biomass can be produced from a given area and the cost associated with operation can be proportionally reduced, although true economic advantage can be fairly judged only by careful comparison of the reduced operation cost with the increased cost for capital investment. The increase in capital cost is mainly determined by the cost of the cover, while the other costs such as increased labor for the installation are relatively negligible. If the cover is made using thin (~0.1mm) polyethylene terephthalate (PET) film (~\$1 m^{-2}) with long lifetime, \$1/sin(θ_v) = \$3.86 is additionally consumed for the pond installation per square meter for the vertex of 30°, increasing the total capital invest by 20 % and biomass price by \$30 ton^{-1}. However, owing to the doubled productivity, total cost per biomass is significantly reduced in this system and therefore the total biomass price from \$536 ton^{-1} to \$277 ton^{-1}, clearly supporting the economic viability. When considering the aspect of mechanical durability and stability, instead of material cost, the cover can be made using thicker films or more permanent materials. While the use of thicker PET or cellulose acetate can be one of the low-cost choices (~\$3 m^{-2}), the use of rigid acryl or glass may add more than \$10 m^{-2} per cover area. With the material cost of \$10 m^{-2}, the economic merit may not be seen for all the vertex angles; however, that system is still worth considering if the land price, which is ignored in Figure S6, is taken into account. For cultivation near big cities, land price takes large portion in the capital cost and high density photosynthesis is truly required. Costly photobioreactors[11] are typically used for such purposes; our approach would achieve high density photosynthesis at much lower cost comparable to open-pond.

Although we assumed the constant operating cost for the systems with and without transparent covers

in Figure S6, the systems implementing the covers are likely to offer additional economic advantages. For example, due to the low insolubility in water, a large amount of CO_2 (~0.05 kg m^{-2} day^{-1} was assumed in Figure S6) is irreversibly lost and it takes 17 % for the total price. The use of a physical cover on top of the open pond serves as a cheap yet effective solution to it. With it considered, the biomass price can be further reduced to \$234 ton^{-1} for the V-shaped system with a vertex of 30° and material cost of \$1 m^{-2} and \$503 ton^{-1} for that with \$10 m^{-2}. Besides, to limit water loss through evaporation and air-borne contamination are of great value, both of which were not taken into consideration in Figure S6. It is true these additional advantages could also be achieved by simple flat covers, but the economic benefits becomes possible only with the V-shaped geometry. (Figure S6) In the manuscript, we showed that the V-shaped system enabled to reach higher final cell density with a shallower depth owing to the intended light trapping effect. The denser the final cell density is, the less energy the subsequent dewatering step consumes.

V-shaped bioreactors used in the experiment

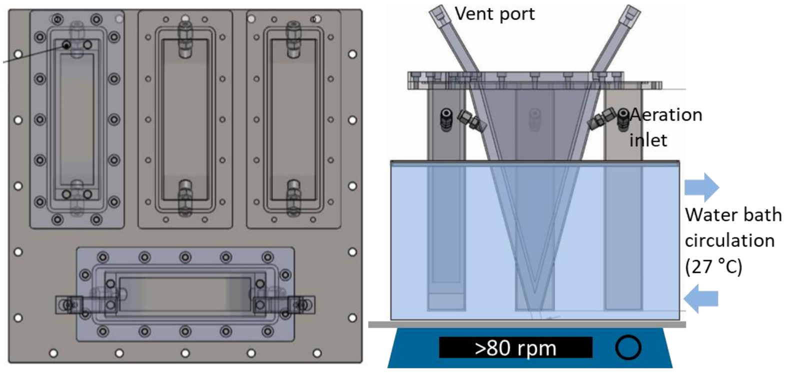

Figure S7. The custom-designed bioreactors used in the experiment for the V-shaped cultivation in the top- and front-view.

We constructed the V-shaped bioreactors cluster to conduct the microalgal cultivation with different volumes simultaneously. The V-shape was formed by covering a V-shaped transparent cover in a V-shaped reactor. The cultivation volume of each reactor was controlled by inserting frames with different height between the top cover and bottom reactor. The light-absorbing apertures were positioned at the top of the reactor to control the illumination area and exclude the possible photon flux from outside. The temperature was controlled by circulating water in the bath in which the reactors were immersed. The air was supplied through two aeration ports with equal rate to balance the environmental condition of both sides of the V-shaped reactor.

References

1 Kim, K. *et al.* High-resolution three-dimensional imaging of red blood cells parasitized by Plasmodium falciparum and in situ hemozoin crystals using optical diffraction tomography. *Journal of Biomedical Optics* **19**, 011005-011005, doi:10.1117/1.jbo.19.1.011005 (2014).

2 Kim, K. *et al.* Optical diffraction tomography techniques for the study of cell pathophysiology. *Journal of Biomedical Photonics & Engineering* **2**, 020201 (2016).

3 Torzillo, G., Sacchi, A., Materassi, R. & Richmond, A. EFFECT OF TEMPERATURE ON YIELD AND NIGHT BIOMASS LOSS IN SPIRULINA-PLATENSIS GROWN OUTDOORS IN TUBULAR PHOTOBIOREACTORS. *Journal of Applied Phycology* **3**, 103-109, doi:10.1007/bf00003691 (1991).

4 Torzillo, G., Sacchi, A. & Materassi, R. TEMPERATURE AS AN IMPORTANT FACTOR AFFECTING PRODUCTIVITY AND NIGHT BIOMASS LOSS IN SPIRULINA-PLATENSIS GROWN OUTDOORS IN TUBULAR PHOTOBIOREACTORS. *Bioresource Technology* **38**, 95-100, doi:10.1016/0960-8524(91)90137-9 (1991).

5 Ogbonna, J. C. & Tanaka, H. Night biomass loss and changes in biochemical composition of cells during light/dark cyclic culture of Chlorella pyrenoidosa. *Journal of Fermentation and Bioengineering* **82**, 558-564, doi:10.1016/s0922-338x(97)81252-4 (1996).

6 Grobbelaar, J. U. & Soeder, C. J. RESPIRATION LOSSES IN PLANKTONIC GREEN-ALGAE CULTIVATED IN RACEWAY PONDS. *Journal of Plankton Research* **7**, 497-506, doi:10.1093/plankt/7.4.497 (1985).

7 Cho, C. & Lee, J. Y. Multi-scale and angular analysis of ray-optical light trapping schemes in thin-film solar cells: Micro lens array, V-shaped configuration, and double parabolic trapper. *Opt. Express* **21**, A276-A284 (2013).

8 Cho, C. *et al.* Random and V-groove texturing for efficient light trapping in organic photovoltaic cells. *Sol. Energy Mater. Sol. Cells* **115**, 36-41 (2013).

9 Cho, C. *et al.* Toward Perfect Light Trapping in Thin-Film Photovoltaic Cells: Full Utilization of the Dual Characteristics of Light. *Advanced Optical Materials* **3**, 1697-1702, doi:10.1002/adom.201500471 (2015).

10 Davis, R., Markham, J. & Humbird, D. Process Design and Economics for the Production of Algal Biomass: Algal Biomass Production in Open Pond Systems and Processing Through Dewatering for Downstream Conversion. (National Renewable Energy Laboratory, 2016).

11 Amer, L., Adhikari, B. & Pellegrino, J. Technoeconomic analysis of five microalgae-to-biofuels processes of varying complexity. *Bioresource Technology* **102**, 9350-9359, doi:10.1016/j.biortech.2011.08.010 (2011).
